# Gold Nanoparticles Conjugated with Dendrigraft Poly-L-lysine and Folate-Targeted Poly(ethylene glycol) for siRNA Delivery to Prostate cancer

**DOI:** 10.7150/ntno.79050

**Published:** 2023-01-09

**Authors:** Georges Minassian, Esther Ghanem, Roland El Hage, Kamil Rahme

**Affiliations:** 1 Department of Sciences, Faculty of Natural and Applied Sciences, Notre Dame University - Louaize, Lebanon.; 2 Faculty of Sciences, Fanar Campus, Chemistry & Physics Department, and Doctoral School, Lebanese University, Beirut, Lebanon.; 3 School of Chemistry & AMBER Centre, University College Cork, Cork, T12 YN60, Ireland.

**Keywords:** gold nanoparticles, polymers, stabilization, siRNA delivery, cancer

## Abstract

Dendrigraft Poly-L-Lysine (d-PLL) coated gold nanoparticles (AuNPs) were synthesized by reducing Tetrachloroauric acid with ascorbic acid in the presence of d-PLL. AuNPs-d-PLL formed a stable colloidal solution that absorbs light at a maximum wavelength (λ_max_) centered at 570 nm as demonstrated by UV-visible (UV-Vis) spectroscopy. From Scanning Electron Microscopy (SEM) analysis, AuNPs-d-PLL were spherical in shape with a mean diameter of 128 ± 47 nm. Dynamic Light scattering (DLS) analysis of the colloidal solution exhibited one size distribution with a hydrodynamic diameter of about 131 nm (size distribution by intensity). Zeta potential (ξ) measurements revealed positively charged AuNPs-d-PLL with ξ about 32 mV, an indicator of high stability in an aqueous solution. The AuNPs-d-PLL was successfully modified with either thiolated poly (ethylene glycol) SH-PEG-OCH_3_ (M_w_ 5400 g mol^-1^) or folic acid-modified thiolated poly (ethylene glycol) SH-PEG-FA of similar molecular weight as demonstrated via DLS and Zeta potential measurements. Complexation of PEGylated AuNPs-d-PLL with siRNA was confirmed by DLS and gel electrophoresis. Finally, we analyzed the functionalization of our nanocomplexes with folic acid via targeted cellular uptake to prostate cancer cells using flow cytometry and LSM imaging. Our findings implicate the broader applicability of folate-PEGylated AuNPs in siRNA-based therapeutics against prostate cancer and perhaps other types of cancer.

## Introduction

Prostate cancer is the second leading cause of death in men despite of innumerable research studies and clinical applications using myriads of anti-cancer drugs and radioactive agents 1-3. The emergence of nanomedicine has diverged from the route of chemotherapy to consider molecular therapies as promising agents to combat resistant tumors and untreatable diseases 4, 5. Potential molecules to treat diseases include small interfering ribonucleic acid (siRNA), also known as short interfering RNA, or silencing RNA molecules that can selectively down-regulate genes implicated in the pathology of the disease 6-8. Due to enzymatic degradation, renal clearance, and electrostatic repulsion with the negatively charged phospholipidic membrane, unprotected siRNA exhibits a short plasma half-life 9, 10. Therefore, the complexation of siRNA with nanoparticles (NPs) has been recently exploited to improve siRNA-based therapeutic applications in cancer treatment 9, 11. In fact, it has been demonstrated that NPs-siRNA cargos can shield naked siRNA from degradation and can be further modified with a ligand to specifically recognize a tumor cell with targeted siRNA delivery 12, 13. NPs capped with positively charged molecules show increased interactions with cellular membranes, enhancing their endocytosis 14, 15. Moreover, besides their particular size and shape-dependent optical properties, gold NPs (AuNPs) stand out as vehicles to shuttle siRNA as they exhibit low cytotoxicity and high affinity to thiols and amines that facilitate their functionalization with biomolecules containing such groups (i.e., antibodies, proteins, and thiol terminated ligands) 16, 17. AuNPs proved to be useful tools in many biomedical applications such as bioimaging, photothermal therapy, and drug delivery 18, 19. For successful use in siRNA delivery, AuNPs should exhibit a positively charged surface to complex the negatively charged siRNA. It has been previously demonstrated that non-toxic, surfactant-free, and positively charged AuNPs could be obtained using _L_-cysteine and polyethyleneimine (PEI) as stabilizers 17, 20. PEI is now widely used in the synthesis of NPs and the delivery of siRNA, since it was shown that a decrease in the pH of cells allows PEI to become protonated, hence facilitating endosomal escape and destabilization of endosomal membranes 21-23. Previously, it was reported that AuNPs-PEI 2KD and AuNP-PEI 25KD can both complex siRNA; however, only PEI-25KD delivers siRNA into PC-3 cancer cells (~98%) 14. Interestingly, the low uptake of AuNPs-PEI 2KD could be enhanced by modification with a targeting ligand (such as anisic acid (AA) or folic acid (FA)) 17, 23. In fact, Au-PEI-AA mediated efficient siRNA uptake into PC-3 prostate cancer cells via binding to sigma receptors 23. Similarly, AuNPs-PEI-FA specifically delivered siRNA into prostate cancer cell lines via binding to folate receptors. Normal cells and tissues have limited expression of folate receptors while most cancer cell lines overexpress folate receptors to enhance their proliferation 24, 25. This resulted in enhanced endogenous gene silencing compared to the non-targeted AuNPs-PEI 17. However, AuNPs-PEI-FA size increased by threefold after siRNA complexation, mainly due to some flocculation. Therefore, the novelty of this study is integrating PEGylation into the nanocomplex model in the presence or absence of folic acid to control AuNP's size, dispersion, and delivery. Also, before PEGylation, we used dendrigraft poly-L-lysine (d-PLL) to stabilize AuNPs in water. To prepare a narrow-molecular-weight distribution of two P-LL (P2) polymers, we followed the same procedure reported by Collet et al. 26. Furthermore, it has been demonstrated that four distinct generations of d-PLL can be obtained via the slow hydrolysis of the side chain-protected building block Lys (Tfa)-NCA 27. However, second-generation equivalents, particularly the guanidinylated derivatives, were found to be an interesting candidate for medication and gene delivery due to their biodegradable nature 28. AuNPs-d-PLL, with a diameter of about 128 nm, were obtained by one-pot chemical reduction of Tetrachloroauric (III) acid trihydrate (HAuCl_4_.3H_2_O) with _L_-ascorbic acid in the presence of d-PLL. The obtained AuNPs-d-PLL were further modified with SH-PEG_5000_-OCH_3_ (Mw 5400 g.mol^-1^) or SH-PEG_5000_-FA as confirmed by DLS and Zeta potential measurements. Compared to PEGylated AuNPs-d-PLL, folate-PEGylated NPs showed enhanced specificity and delivery of siRNA to PC-3 PSMA cells. Thus, our nano-formulation proves to outperform existing models in the literature and can be customized with any siRNA to target PC or other types of cancer overexpressing folate receptors.

## Materials and Methods

### Chemicals

Purified H_2_O (resistivity of 18.2 MΩ cm) was used as a solvent. Glassware was cleaned with aqua regia (3 parts of concentrated HCl and 1 part of concentrated HNO_3_), rinsed with distilled water, ethanol, and acetone, and oven-dried before use. Tetrachloroauric (III) acid trihydrate 99% (HAuCl_4_, 3H_2_O), _L_-Ascorbic Acid, Phosphate Buffer Saline (PBS), Folic Acid (FA), N-hydroxy succinimide (NHS), *N, N'*-Dicyclohexylcarbidiimide (DCC), Triethylamine (TEA), Dimethyl sulfoxide (DMSO), and MISSION® siRNA Fluorescent Universal Negative Control #1, 6-FAM were purchased from Sigma Aldrich. Thiol-terminated poly (ethylene glycol) methyl ether (SH- PEG_5000_-OCH3), M_w_ = 5400, and SH-PEG-NH_2_ M_w_ = 5000 were purchased from Polymer Source.

Poly (_L_-lysine) dendrigraft (PLL) with an average molecular weight of 7 KDa was synthesized and characterized in a previously published work 26. All chemicals were used without further purification or modification.

### Cells and Culture Conditions

Prostate cancer cell lines, namely wild-type PC-3 WT (PSMA negative) and PC-3 PSMA positive cell lines, were kindly provided by Prof. Giulio Fracasso (University of Verona, Italy). Cell lines were cultured in RPMI 1640 (Sigma- Aldrich) and complemented with 10% fetal bovine serum and antibiotics (100 U/mL penicillin and 100 μg/mL streptomycin) in a humidified atmosphere with 5% CO_2_ at 37 °C. Cells were grown in a monolayer (70-80% confluency), before being transfected with our AuNPs formulations: siRNA.AuNPs-d-PLL-PEG-OCH_3_ and siRNA.AuNPs-d-PLL-PEG-FA.

### siRNA Transfection

For easy visualization and assessment of siRNA transfection efficiency, MISSION® siRNA Fluorescent Universal Negative Control #1, 6-FAM was used. According to the manufacturer's instructions, siRNA was dissolved in 1mL sterile DNAse/RNAse free water (Sigma-Aldrich). Cells were then transfected with 100 µL siRNA (25 and 50 nM) coupled with AuNPs-d-PLL-PEG-OCH_3_ or AuNPs-d-PLL-PEG-FA (500 µg/mL) for four hours at room temperature. Cells were then washed and prepped for confocal microscopy.

### Synthesis and PEGylating of positively charged d-PLL-Gold (Au) Nanoparticles

***Synthesis of AuNPs-d-PLL***: In a 50 mL round flask, 2 mL HAuCl_4_ (12.5 mM) was added under stirring to 21.36 mL of deionized water. This was followed by the quick addition of 1.250 mL of d-PLL (200 mM) as a coating ligand and 0.39 mL of ascorbic acid (0.1 M) as a reducing agent. Upon adding ascorbic acid, the color of the solution changed from pale yellow to blue-reddish/brown. The solution was kept under stirring overnight. The obtained nanoparticles were analyzed by SEM, DLS, and Zeta potential measurements.

***Synthesis of AuNPs-PEG by PEGylation of AuNPs-d-PLL***: 0.27 mL of SH-PEG 5000 (185 mM) was added dropwise for 5 minutes to 5 mL of AuNPs-d-PLL colloidal solution under stirring condition for two hours. The PEGylation grafting was confirmed by DLS and Zeta potential measurements.

***Synthesis of AuNPs-PEG-FA by grafting SH-PEG-Folate to AuNPs-d-PLL***: To 5 mL of AuNPs-d-PLL colloidal solution under stirring, 0.27 mL of a pre-synthesized SH-PEG-FA 5000 (185 mM) was added dropwise for 5 minutes, and the solution was kept under stirring for two hours. SH-PEG-FA coating was confirmed by DLS and Zeta potential measurements.

Finally, all nanoparticle formulations were centrifuged at 10,000g for 15 mins to collect the particles. Their supernatant was discarded and the final NP product was resuspended with 50 mL deionized water to remove any unreacted materials from the mixture.

### Synthesis of SH-PEG-FA

The thiolated poly (ethylene glycol) folate (SH-PEG_5000_-FA) was obtained by coupling SH-PEG-NH_2_ (Mn 5,000) with Folate N-hydroxysuccinimidyl ester via a previously published method 29-31. Briefly, folic acid (1.5 g, 3.398 mmol) and TEA (0.45 mL, 5.01 mmol) were dissolved in DMSO (45 mL use the minimum DMSO) and DCC (0.84 g, 4.08 mmol) was slowly added to it. Afterward, the solution was stirred for 1-h at room temperature in the dark; then NHS (0.48 g, 4.08mmol) was added in 3 mL DMSO. The mixture was mixed overnight in the dark at room temperature. Then the solution was filtered to remove the insoluble byproduct dicyclohexylurea, and the filtrate was precipitated using diethyl ether. FA-NHS-Ester was collected by filtration, washed with dry THF, and left to dry under vacuum overnight. The final FA-NHS was stored at - 20°C for further use. Activated folic acid FA-NHS was reacted with SH-PEG-NH2 to produce SH-PEG-Folate. In brief, FA-NHS (0.15g, 0.28 mmol) and SH-PEG-NH2 (1g, 0.2 mmol) were dissolved in 6 mL DMSO. The mixture was left under stirring for 48 h in the dark at room temperature. Afterward, chloroform was added to the reaction mixture and the solution was collected by filtration. The obtained solution was precipitated using diethyl ether and SH-PEG-FA was collected by centrifugation and left to dry under vacuum. The formation of SH-PEG-FA was confirmed by Fourier-transform infrared spectroscopy (FTIR) and UV-vis spectroscopy.

### Dynamic Light Scattering and Zeta Potential measurements

The size distribution and surface charge (zeta potential) of AuNPs colloidal solutions were determined by DLS with the Malvern Zetasizer Nano-ZS (model ZEN3600; Malvern, Worcestershire, WR14 1XZ, UK) using the default NIBS 173° back scatter technique. The model used in the fitting procedure was based on Mark Houwink parameters. The cumulative fit provided by the vendors was used to treat the data. Using disposable folded capillary cuvettes, measurements were made on pristine solutions of AuNPs (~50 µg/mL) at 25 ^ᵒ^C. Triplicates of each sample were recorded to facilitate result comparison.

### UV-Vis and SEM analysis of various AuNPs

0.5 mL of various AuNPs-d-PLL formulations was withdrawn and injected into black quartz cuvettes for UV-Vis spectroscopy. Purified H_2_O was used as a blank and readings were performed in triplicates using UV-Vis Analytikjena SPECORD® 250 PLUS spectrophotometer (300-900-nm range, 0.5 nm resolution). Hitachi S-4300 environmental scanning electron microscope (ESEM) operated at 12.5 kV. Image J software was used to characterize the size and distribution of the particles.

### Fourier transform infrared spectroscopy (FTIR)

The Infrared spectra are recorded on a Perkin Elmer (Spectrum Two) FT-IR spectrometer with a resolution of 1 cm^-1^ and a scanning range of 400 cm^-1^-4000 cm^-1^.

### Gel Electrophoresis

In 200 µL DNAse/RNAse free PCR tubes, AuNPs:siRNA with three different mass-to-ratio (MR) concentrations were prepared (MR 10, 20, and 30). siRNA concentration was set at 0.25 µg for all samples. For MR 10, 2 µL siRNA (13.8 µg) was added to 5.52 AuNPs in 11.04 µL RNAse-free water. For MR 20, 2 µL siRNA (13.8 µg) followed by 11.04 µL AuNPs and 5.52 µL RNAse-free water was added. For MR 30, 2 µL siRNA (13.8 µg) was mixed with 16.56 µL AuNPs. All samples were kept in the dark at RT for 1hr with constant low shaking, followed by adding 2 µL loading buffer. Eventually, samples were loaded on a previously prepared 1.5 % Agarose gel and left to run at 100 mV for 30 mins. The obtained gel was photographed under UV light using ChemiDoc™ and the intensity of bands was analyzed.

### WST-1 Cell Proliferation Assay

PC-3 PSMA cells at the log phase were seeded at 5 × 10^4^ cells/mL and incubated overnight. Cells were treated with 50 µg/mL of AuNPs-d-PLL-PEG-FA between 4 to 24 hours and tested at various time points. For control, AuNPs were resuspended with deionized water and serum-free media to reach the desired concentration in a final volume of 500 µL. Out of which 200 µL were loaded into the respective wells. After each time point, cells were washed with 1x PBS and incubated with 10 µL of WST 1 reagent in serum-free media for 2 hours 32. The tetrazolium salt, WST-1, is cleaved by a cellular mechanism that occurs mainly at the cell surface, forming soluble formazan 32. Results were read at 450 nm using the Thermofisher MultiGo-Scan ELISA reader.

### Flow Cytometry

PC-3 PSMA cells were seeded at 100,000 cells per well in a flat bottom adherent 24-well plate (n = 3) and cultured for 24hrs following normal growth conditions (RPMI, 5% CO_2_, 37 °C). Cells were then incubated with 100 µL of 25 and 50 nM fluorescent siRNA complexed with AuNPs-d-PLL-PEG MR 25 (500 µg/mL) and AuNPs-d-PLL-PEG-FA MR 25 (500 µg/mL) and incubated for 24hrs in 300 µL normal growth medium (RPMI + 10 % FBS + 0.1% PS). Cells were then washed twice with culture grade 1x PBS and trypsinized. Collected cells were centrifuged at 1000 rpm for 5 mins and resuspended in 1mL cold FACS Buffer (3 % PFA + 0.2 % BSA, in 1x PBS). Gates were set at 50,000 cells per sample, and siRNA-FITC fluorescence was detected using a Partec Cube 8 Flow Cytometry and analyzed using FlowJo.

### Fluorescence microscopical analysis of siRNA-NPs localization

PC-3 PSMA cells were seeded at 1x10^6^ cells per well in a flat bottom adherent 6-well plate and incubated for 24hrs following normal growth conditions (RPMI, 5% CO_2_, 37 °C). Cells were then transfected with 100 µL of 25 and 50 nM fluorescent siRNA complexed with AuNPs-d-PLL-PEG MR 25 (500 µg/mL) and AuNPs-d-PLL-PEG-FA MR 25 (500 µg/mL) and incubated for four hours in 500 µL serum-free RPMI. After two washes with 1x PBS, cells were fixed with 4% PFA for 10 mins at room temperature in the dark. Following fixation, cells were washed again with 1x PBS and incubated with 1% Triton X 100 in PBS for 5 mins. Cells were then blocked for 30 mins using a solution of PBS-Tween 20, BSA, and Tris pH 8.8. Cells were then washed and incubated with monoclonal rabbit anti-Early Endosomal Marker (EPR4245; dilution 1:100; Alexa Fluor® 647) and rabbit monoclonal anti-Lysosome Membrane Marker (EPR6599; dilution 1:150; Alexa Fluor® 594). Before mounting, nuclear fragmentation was tracked with 100 µL DAPI (1.5 µg/ml; Vector Laboratories, Burlingame, CA, USA) at 4 °C for 1 hr. Images were obtained by laser scanning confocal microscopy, Zeiss LSM 700. For DAPI, UV-light with a blue filter was used.

### Testing Co-localization using Z-stacks

Z-stack slicing was performed on representative samples of AuNPs-d-PLL-PEG.siRNA 50 nM and AuNPs-d-PLL-FA.siRNA 50 nM. Laser gain was set at 600 nm for Alexa Fluor 488, EEA-1, and M6RP, and 500 nm for the DAPI stain. In total, 20 sections were captured with a focal distance separation of 10 µm at an interval of 1 frame/second. The smallest section was set at 2.73 µm and all lasers had matched pinholes.

### Statistical Analysis

Data are reported as means ± SEM and analyzed by one-way ANOVA. The differences between experimental and control groups were assessed by post-hoc and Tukey's tests. Statistical significance was recognized at *p* < 0.05 and each experiment was conducted and validated at least three times. Significance was reported on each graph with * representing a *p-value* < 0.05, ** representing a *p-value* < 0.01, and *** representing a *p-value* < 0.001. *NS* corresponds to a non-significant difference.

## Results

### Characterization of the pristine solution of AuNPs-d-PLL

**Figure 1:**
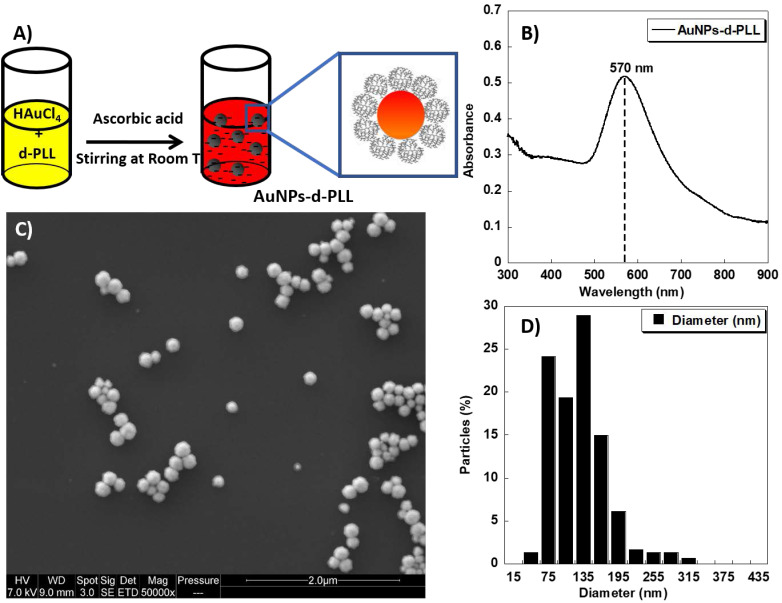
** A)** Schematic model of the one-pot synthesis of AuNPs-d-PLL solution designed in this study.** B)** UV-visible spectrum of the obtained AuNPs-d-PLL colloidal solution. **C)** SEM image of the AuNPs-d-PLL (scale bar 2 µm); and **D)** Histogram showing the size distribution of the AuNPs-d-PLL from *Image J* analysis.

AuNPs-d-PLL aqueous colloidal solution was obtained via a simple one-pot reduction of tetra chloroauric acid with ascorbic acid in the presence of d-PLL with an average molecular weight of 7 KDa as a capping agent (Figure 1A). The resulting AuNPs-d-PLL formed a stable colloidal solution with a visible absorption spectrum at a maximum wavelength (λ_max_) centered at 570 nm as demonstrated by UV-visible spectroscopy (Figure 1B). As shown in Figure 1C, SEM image demonstrates uniform AuNPs-d-PLL with a nearly spherical shape and a mean diameter of about 128 ± 47 nm as analyzed by *Image J* software (Figure 1D).

Furthermore, the successful coating of AuNPs with d-PLL during the reduction process was confirmed by the positive value of their zeta potential +32 mV as depicted in Table 1. This is most likely due to the adsorption of d-PLL onto AuNPs and indicates a high stability in water.

### FTIR analysis of the NHS-activated folic acid (FA-NHS), SH-PEG-NH_2,_ and SH-PEG-FA

**Figure 2:**
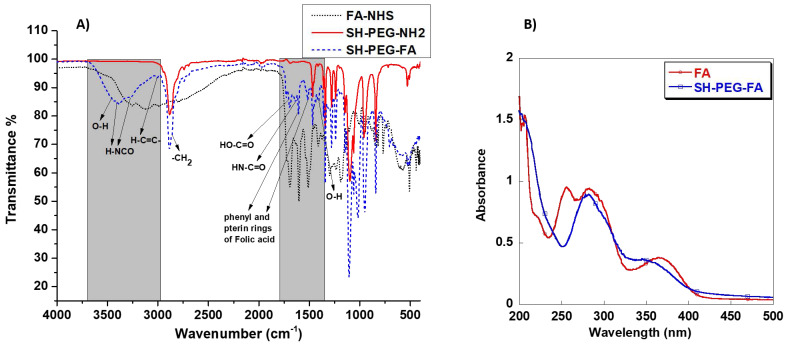
** A)** FTIR analysis of the NHS-activated folic acid (FA-NHS) dot line, SH-PEG-NH_2_ solid line, and SH-PEG-FA dash line. **B)** UV-visible spectra of Folic acid (○) and SH-PEG-FA (□).

The attachment of folic acid to SH-PEG-NH_2_ via NHS/DCC coupling was confirmed by FTIR and UV-vis spectroscopy. Figure 2A depicts the results of an FTIR study of the various ingredients utilized to create SH-PEG-FA. Comparative spectra demonstrate the formation of the SH-PEG-FA, confirming the presence of characteristic folic acid peaks, primarily in the O-H (bending), C=C, and C=O (stretching) regions highlighted in Figure 2A between 1350 and 1750 cm^-1^. In addition to the OH and NH stretching region observed in carboxylic acid and amide between 3000 - 3700 cm^-1^. Interestingly, it is evident that the stretching of OH and the N-H in amide show a strong existence at 3100-3500 cm^-1^ in the final product SH-PEG-FA. Furthermore, the vibration of carbonyl in the secondary amide bond at 1634 cm^-1^ formed between the primary amino group of the SH-PEG-NH_2_ and the carbonyl group of Folic-NHS is also observed in the product. Finally, the presence of H-C=C stretching at 3008 cm^-1^ and the presence of C=C at 1606 cm^-1^ and 1511 cm^-1^ is indicative of the presence of (phenyl and pterin rings**)** of folic acid, further confirming the successful attachment of FA to SH-PEG-NH_2_ 33.

Similarly, UV-vis analysis on folic acid and SH-PEG-FA show that the latter exhibited characteristic absorption peaks of folic acid with a slight shift at about 282 nm and 350 nm, confirming the successful attachment of folic acid to SH-PEG-NH_2_, as demonstrated in Figure 2B.

### Characterization of differently coated AuNPs (d-PLL, PEG, PEG-FA) and siRNA complexation

**Figure 3:**
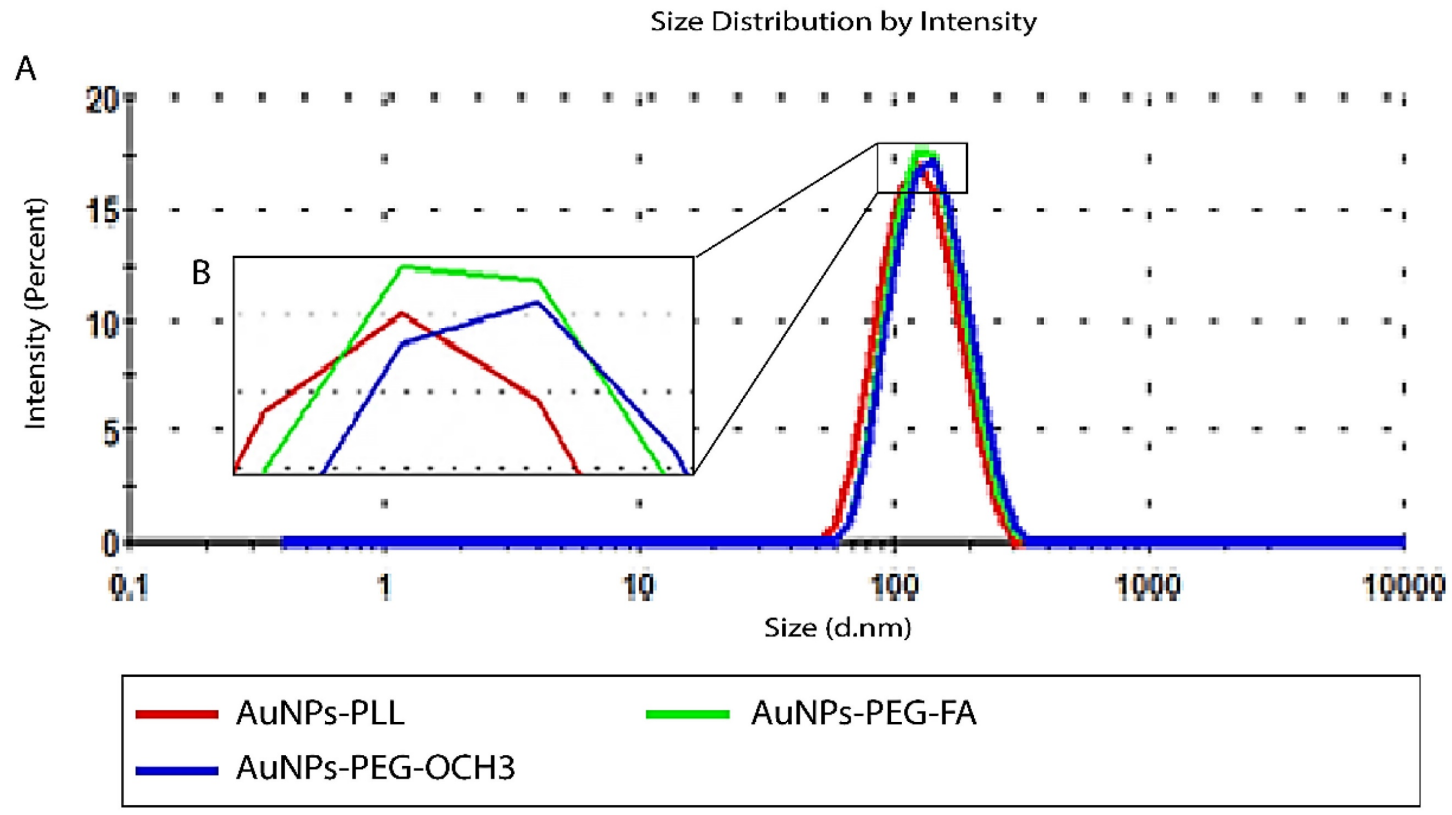
Size distribution by intensity of AuNPs-d-PLL vs. AuNPs-d-PLL-PEG-OCH3, and AuNPs-d-PLL-PEG-FA. Panel A**:** An increase of 7nm in size by intensity was noted when comparing AuNPs-d-PLL in the red curve (118.8 ± 1) to AuNPs-d-PLL-PEG-FA in the green curve (125.5 ± 5). An increase of 15 nm in size by intensity was observed when comparing AuNPs-d-PLL (118.8 ± 1) to AuNPs-d-PLL-PEG-OCH3 in the blue curve (133.1 ± 0.9). Panel B: Inset of enlarged peaks to clarify the variation in size (n = 3).

**Figure 4:**
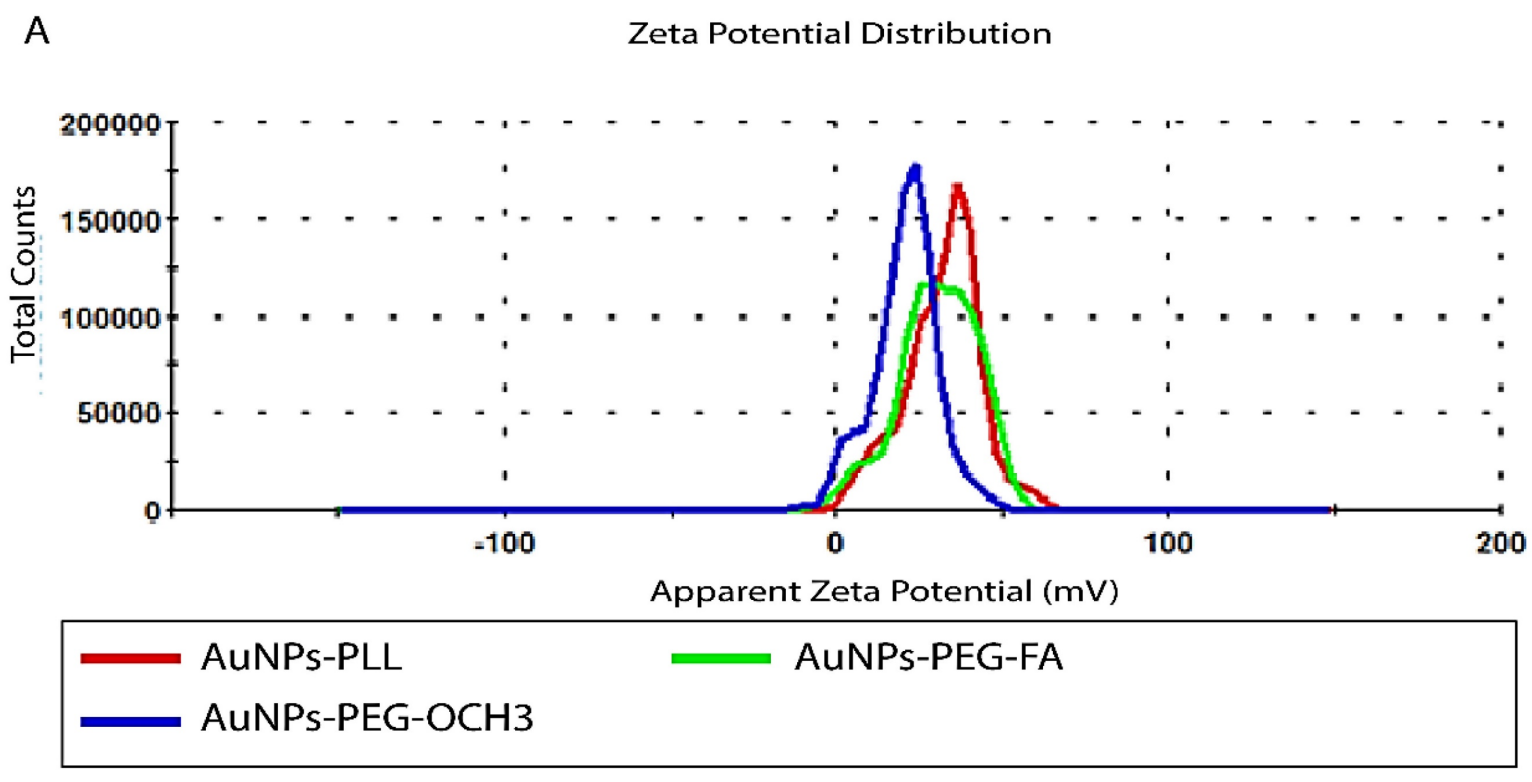
Zeta measurements of AuNPs-d-PLL vs. AuNPs-d-PLL-PEG-OCH3, and AuNPs-d-PLL-PEG-FA. AuNPs-d-PLL-PEG-OCH3 recorded the lowest charge at 21 ± 1 mV when compared to AuNPs-d-PLL 32 ± 1.3 mV, and AuNPs-d-PLL-PEG-FA (30.2 ± 0.9). A 2 mV decrease in zeta potential was observed between AuNPs-d-PLL and AuNPs-d-PLL-PEG-FA. (n = 3).

For successful characterization of the physicochemical properties of SH-PEG-FA, we ought to test the effect of d-PLL coating on the overall charge and size using DLS/Zeta potential measurements and UV-visible spectroscopy. Therefore, AuNPs-d-PLL particles were further modified with folate-targeted (SH-PEG-FA) or untargeted (SH-PEG-OCH_3_) thiolated poly (ethylene glycol). Thiol groups have a high affinity for the surface of AuNPs, facilitating the formation of AuNPs-SH-PEG-FA and AuNPs-SH-PEG-OCH3 complexes. Based on our DLS findings, AuNPs-d-PLL had a hydrodynamic diameter of 118.8 ± 1 nm (Z avg), which increased with the gradual addition of SH-PEG-FA, reaching a size of 125.5 ± 5 nm (Table 1). AuNPs-d-PLL-PEG revealed the largest diameter of 133.1 ± 0.9 nm (Z avg) when compared to the previously mentioned particles in Figure 3. As for the surface charge of AuNP complexes, AuNPs-d-PLL registered a positive charge of +32 ± 1.3 mV, and steadily dropped upon the addition of SH-PEG-OCH_3_ and SH-PEG-FA, reaching +30.2 ± 0.9 for AuNPs-d-PLL-PEG-FA and +21 ± 1 mV for AuNPs-d-PLL-PEG (Table 1, Figure 4).

**Scheme 1:**
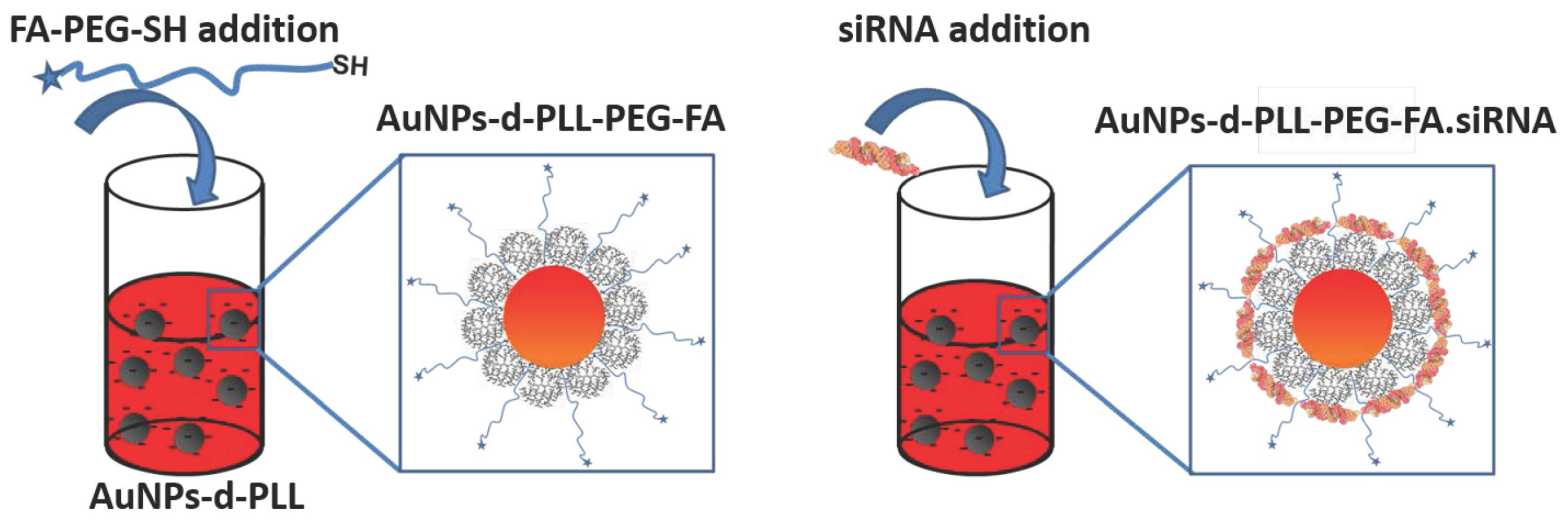
Attachment of SH-PEG-FA onto AuNPs-d-PLL (left) and complexation of siRNA with AuNPs-d-PLL-PEG-FA. A similar method was used for AuNPs-d-PLL-PEG-OCH_3_.

**Figure 5:**
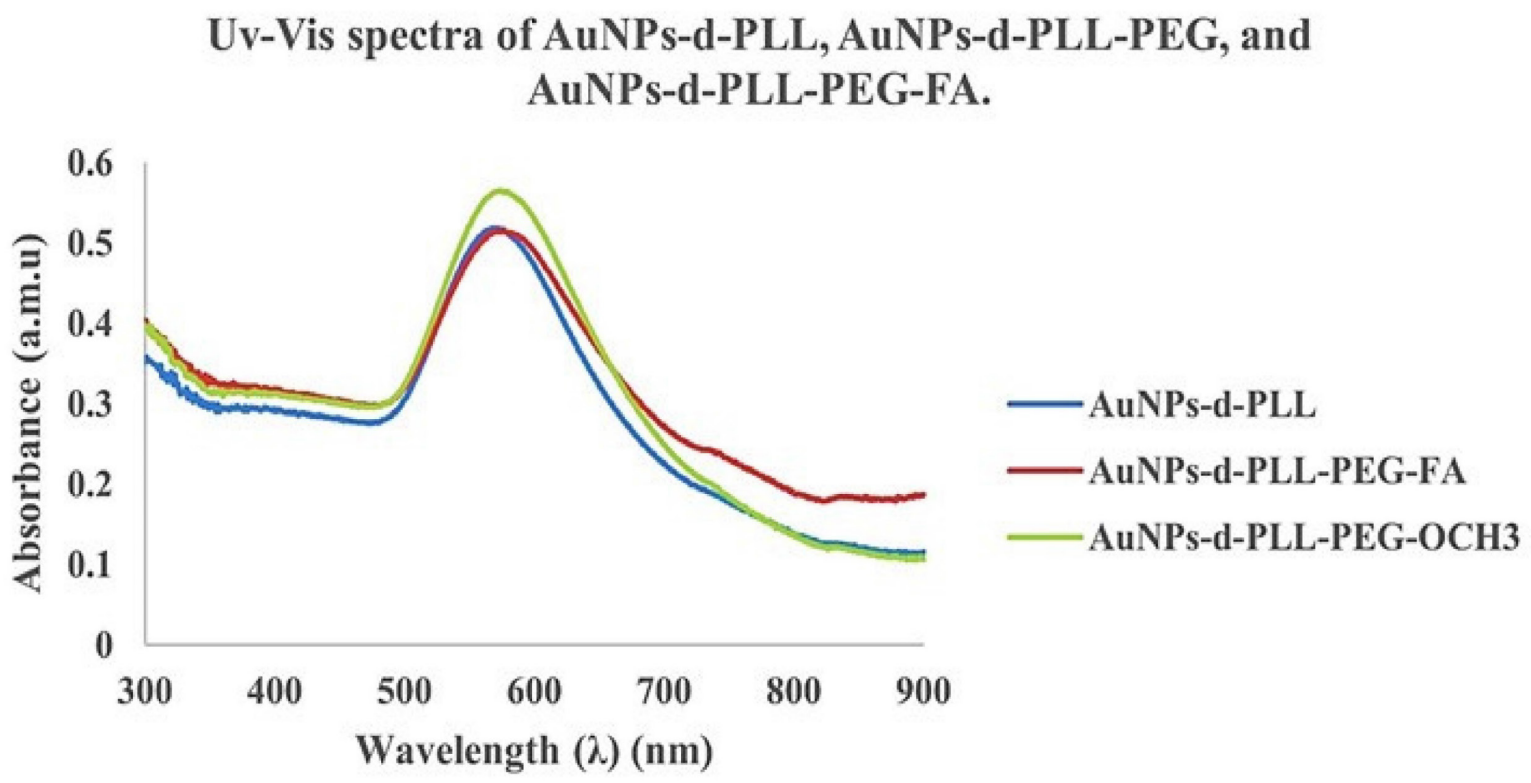
UV-Vis spectra of AuNPs-d-PLL, AuNPs-d-PLL-PEG, and AuNPs-d-PLL-PEG-FA. A clear redshift of 4 nm from 570 to 574 nm (AuNPs-d-PLL-PEG-FA), and of 6 nm from 570 to 576 nm (AuNPs-d-PLL-PEG-OCH_3_) is noted upon the addition of SH-PEG-FA and SH-PEG-OCH3 to AuNPs-d-PLL (n = 3).

The UV visible spectra of the resulting AuNPs-d-PLL-PEG nanoparticles show a clear redshift of about 4 nm from 570 to 574 nm for AuNPs-d-PLL-PEG-FA and 6 nm from 570 to 576 nm for AuNPs-d-PLL-PEG-OCH_3_. A successful attachment of PEG to the surface of the AuNPs was further demonstrated by the absence of any evidence of aggregation (Figure 5). Interestingly, AuNPs-d-PLL-PEG retained a positive surface charge and were able to further complex with siRNA with or without folate, as shown in Scheme 1.

**Figure 6:**
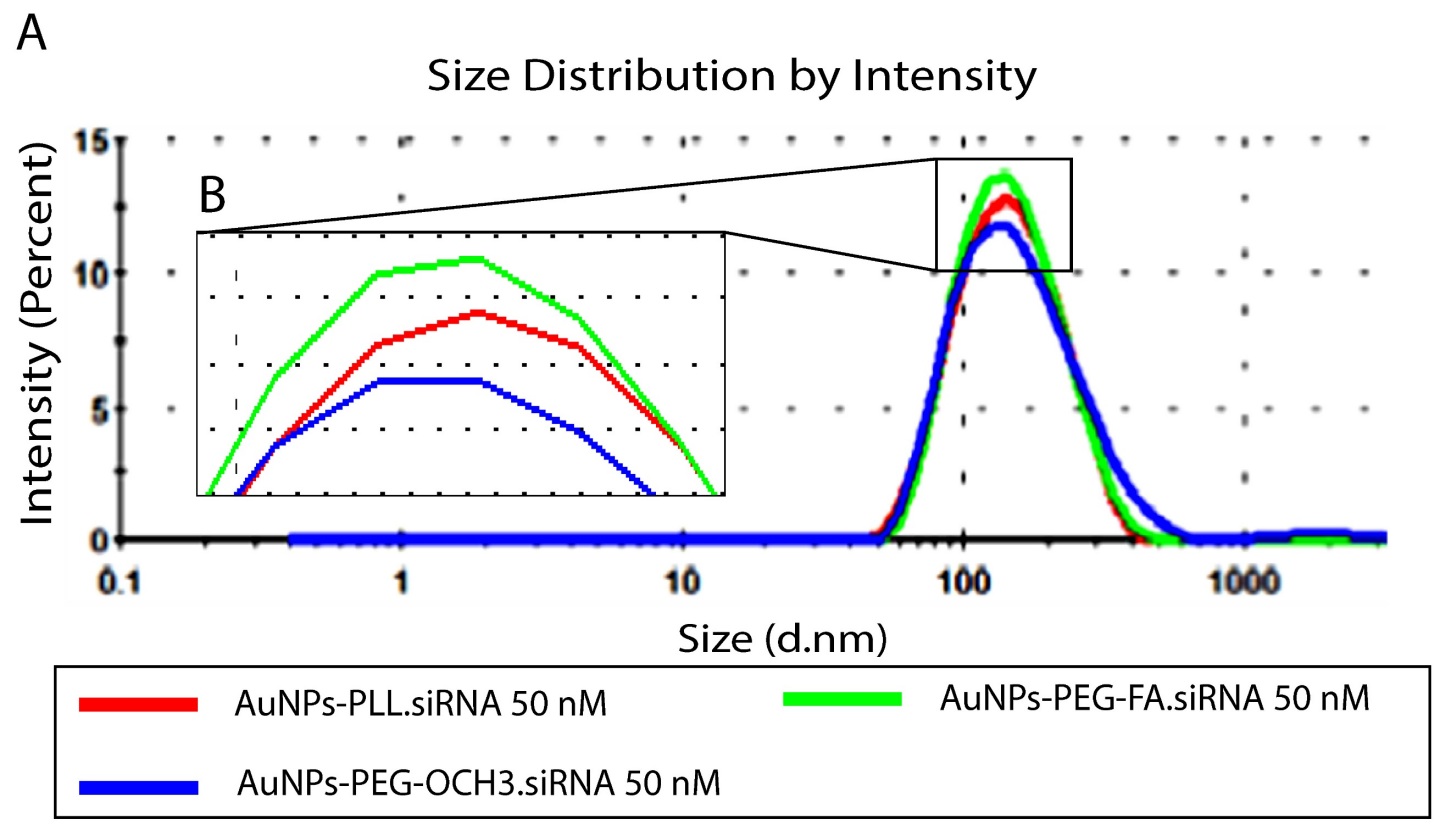
Size distribution by intensity of AuNPs-d-PLL.siRNA 50nM Vs. AuNPs-d-PLL-PEG-OCH3.siRNA 50nM, and AuNPs-d-PLL-PEG-FA.siRNA 50nM. Panel A: An increase in the size of 21 nm was noted for AuNPs-d-PLL.siRNA, as compared to 10.5 nm for AuNPs-d-PLL-PEG-FA.siRNA. Finally, AuNPs-d-PLL-PEG-OCH3 showed an increase in the size of 1 nm only. Panel B: Inset of enlarged peaks to clarify the variation in size. AuNPs:siRNA was maintained at 25 (n = 3).

Characterized AuNPs-d-PLL were then complexed with two concentrations of siRNA (25 and 50 nM), while maintaining a constant MR at 25 of AuNPs.siRNA across all samples. For particles subjected to 25 nM siRNA, AuNPs-d-PLL.siRNA demonstrated an increase in hydrodynamic diameter reaching 123.5 ± 2.2 (Z avg), as seen in Table 2. Furthermore, AuNPs-d-PLL-PEG.siRNA exhibited a Z avg of 135 ± 4 nm compared to AuNPs-d-PLL-FA.siRNA with an average size of 193.5 ± 1.9 nm (Table 2). The greatest change was observed when measuring AuNPs surface charge upon the addition of the very negatively charged siRNA. AuNPs-d-PLL.siRNA 25 nM witnessed a decrease in zeta potential from +32 ± 1.3 mV to -21.13 ± 0.5 mV, followed by PEGylated forms, AuNPs-d-PLL-PEG.siRNA 25 nM, with a charge of -14.13 ± 4.87 mV, and finally the surface charge decreased from +30.2 ± 0.9 mV to reach -11.41 ± 3.78 mV for AuNPs-d-PLL-FA.siRNA 25 nM (Table 2). Subsequently, for particles subjected to 50 nM siRNA, the same analysis was performed and similar results were observed (Table 3). As seen in Figure 6, particles exhibited a slight increase in their average size, without any sign of aggregation. AuNPs-d-PLL.siRNA 50 nM attained the highest Z avg of 139.6 ± 1.3 nm followed by AuNPs-d-PLL-FA.siRNA 50 nM (Z avg of 136 ± 1.5 nm), and finally an average diameter of 134 ± 1 nm for AuNPs-d-PLL-PEG.siRNA 50 nM. Lastly, particles also exhibited noticeable changes in zeta potential. Based on the results in Table 3, AuNPs-d-PLL.siRNA 50 nM became strongly negative with a surface charge of -23.6 ± 2 mV, followed by AuNPs-d-PLL-FA.siRNA 50 nM with a zeta potential of -11.5 ± 1.5 mV, and finally AuNPs-d-PLL-PEG.siRNA 50 nM with a surface charge of -4.4 ± 1.4 mV.

### Toxicity testing of various AuNPs

**Figure 7:**
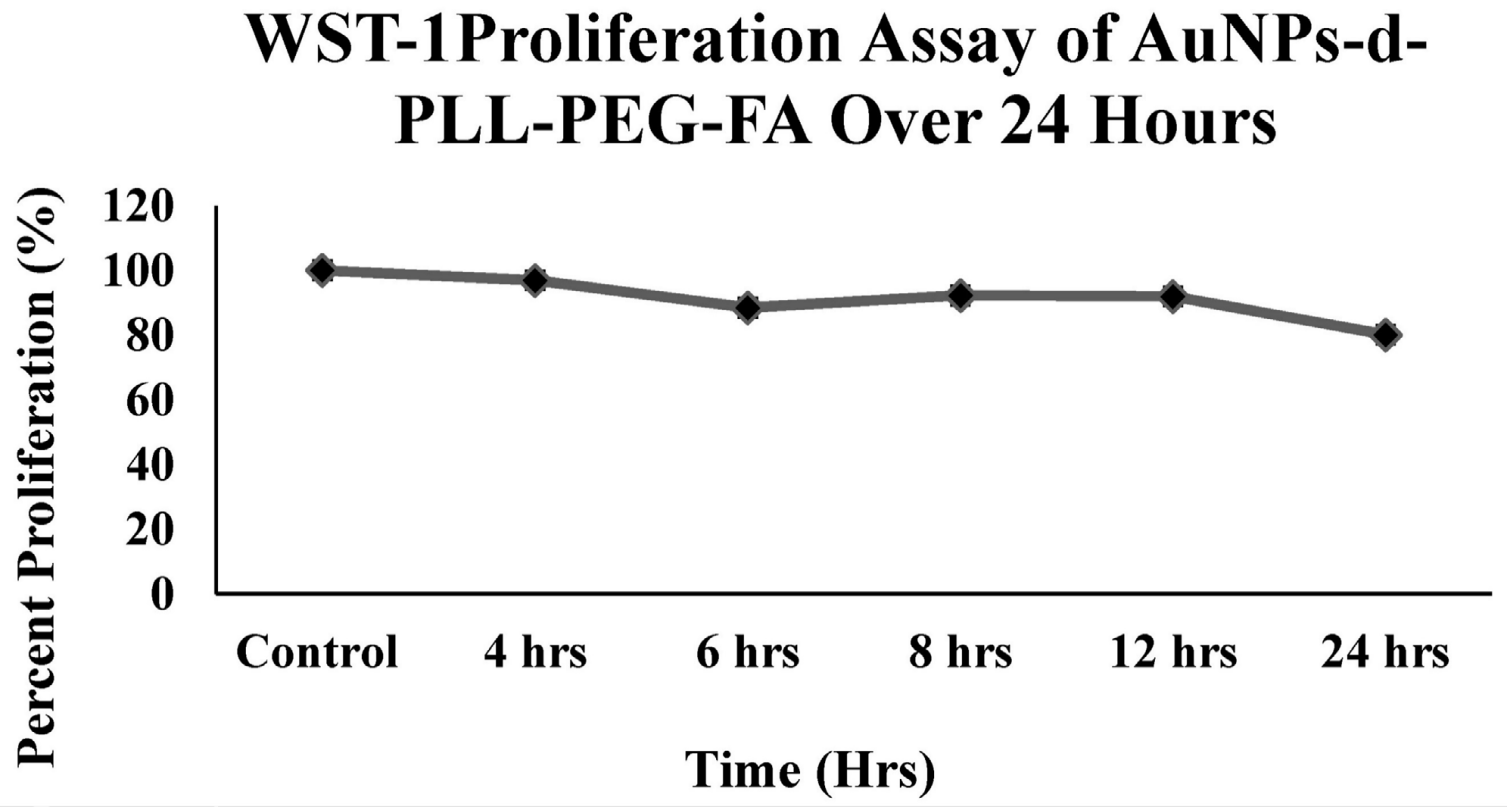
WST-1 Cell Proliferation Assay of AuNPs-d-PLL-PEG-FA. The line chart demonstrates the cytotoxicity profile of AuNPs-d-PLL-PEG-FA when incubated with PC-3 PSMA cells over a period of 24 hours. NP concentration was fixed at 50 µg/mL for all time points. Cells were initially seeded onto a 96-well plated, left to reach confluency, and incubated with 50 µg/mL AuNPs-d-PLL-PEG-FA for 4, 6, 8, 12, and 24 hours. Absorbance was read at 450 nm.

Despite the importance of deciphering the physicochemical properties of our AuNPs to deliver siRNA, what remains at stake is their cytotoxic effect. This is easily tested *in vitro* using colorimetric assays, such as WST-1. WST-1 reduction is highly dependent on the glycolytic production of NAD(P)H in viable PC-3 PSMA cells. Data reported in Figure 7 support the non-cytotoxic effects of AuNPs-d-PLL-PEG-FA (50 µg/mL) since the percent viability remained above 80% for all the selected time points. Even after a 24-hour incubation period, the percent viability remained satisfactory, with around 80% viability as compared to the control.

### Gel Electrophoresis for siRNA complexation of differently coated AuNPs (PLL, PEG, PEG-FA)

**Figure 8:**
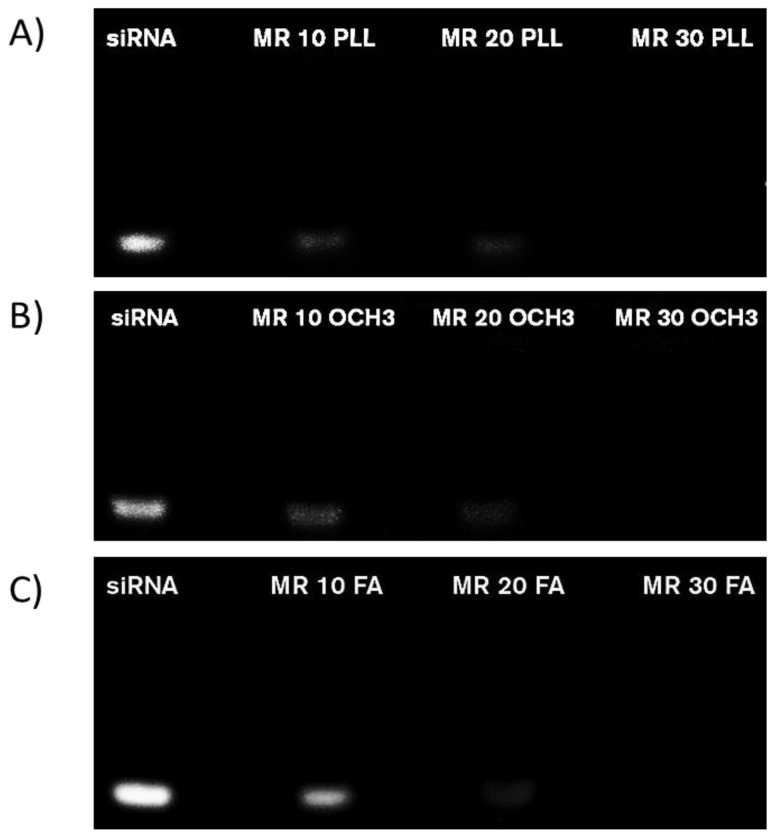
Agarose gel electrophoresis of various AuNPs.siRNA MRs captured under UV light with ChemiDoc™. Free siRNA (0.25 µg/mL) strands were used for the control (first lane in all panels). AuNPs.siRNA complexation was observed on a 1.5 % agarose gel with various coating: AuNPs-d-PLL (A), AuNP-d-PLL-PEG-OCH_3_ (B), and AuNPs-d-PLL-PEG-FA (C) at MRs 10, 20, and 30, respectively. An evident decrease in band intensity was visible at MR 20. At MR 30, the siRNA band entirely disappeared in all three samples indicating a successful and complete complexation (n = 5).

To elucidate the capacity of our NPs to bind siRNA, siRNA complexation was visualized *in situ* on a 1.5% agarose gel. AuNPs-d-PLL.siRNA complexation was initiated at MR 10, with a slight decrease in band intensity at MR 20, followed by complete band disappearance at MR 30, marking total AuNPs-d-PLL.siRNA complexation (Figure 8). On the other hand, AuNP-d-PLL-PEG OCH_3_.siRNA complexation at MR 10 was less evident when compared to AuNPs-d-PLL.siRNA, with a similar decrease in band intensity at MR 20 (Figure 8, panels A and B). Similarly, complete complexation was achieved at MR 30. Finally, AuNP-d-PLL-PEG FA.siRNA complexation in panel C was less noticeable at MR 10 when compared to NPs in panels A and B. However, comparably to NPs from panels A and B, complexation became prominent at MR 20 and finalized at MR 30. The latter indicates that complete siRNA complexation was achieved between MR20 and MR30, irrespective of the coating agent used on our AuNPs. Lastly, positively charged AuNPs-d-PLL (+32 ± 1.3 mV) showed the earliest signs of siRNA complexation with the faintest band intensity at MR 10 (Figure 8, panel A).

### Cellular Uptake of various AuNPs complexes by flow cytometry

**Figure 9:**
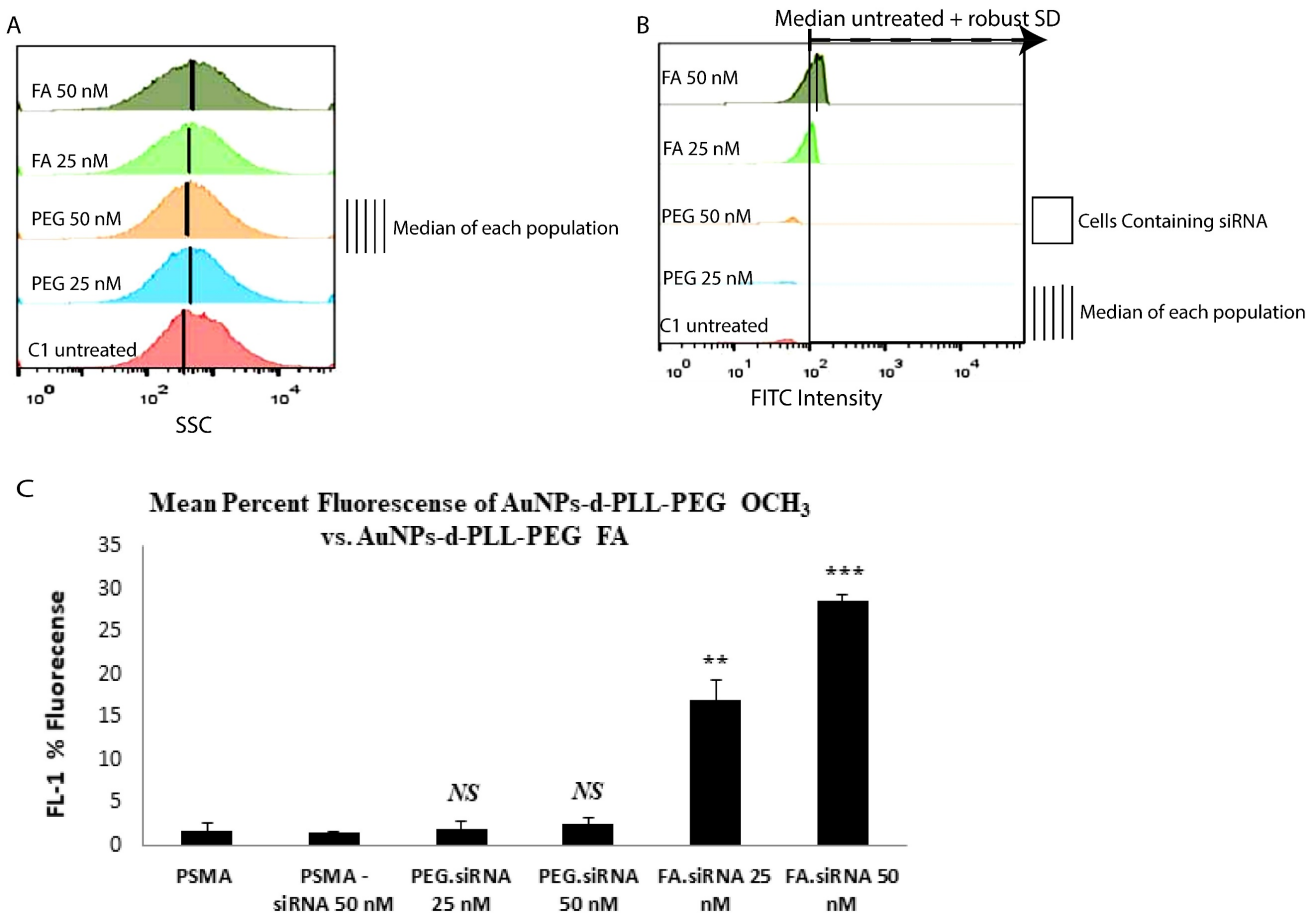
Mean Percent Fluorescence of AuNPs-d-PLL-PEG OCH_3_ vs. AuNPs-d-PLL-PEG-FA. PC-3 PSMA cells and PC-3 PSMA.siRNA (50 nM) were used as control. Cells incubated with siRNA.AuNPs-d-PLL-PEG (25 and 50 nM) showed insignificant fluorescence (≤ 5 %). Cells incubated with siRNA.AuNPs-d-PLL-FA (25 nM) presented a mean percent fluorescence of ~ 17% while increasing siRNA.AuNPs-d-PLL-FA to 50 nM had the highest mean percent fluorescence of ~ 30% (n = 3).

Counts of fluorescently labeled siRNA-PSMA were compared among different doses and types of NPs, as illustrated in Figure 9. Concerning side scattering, no significant change was observed among the samples (panel A). Therefore, quantification of cells containing FITC-labeled-siRNA was performed. Based on a method adopted by Shin et al 2020, the percentage of cells containing FITC siRNA was calculated from cells with higher FITC signals than the threshold 20. This threshold value was calculated from the median + robust SD (retrieved from FlowJo) of the control, untreated samples (panel B), allowing for statistically meaningful measurements. Then, the data was extracted and plotted using Excel (panel C).

In terms of fluorescence, a non-remarkable fluorescent FL-1 intensity corresponds to the FITC siRNA signal as observed for naked siRNA and AuNPs-d-PLL-PEG.siRNA (Figure 9C). On the other hand, increased FL-1 fluorescence was marked for 25 and 50 nM AuNPs-d-PLL-PEG-FA.siRNA, which is quite significant compared to naked siRNA-NPs (~ 17%, and ~ 30%, respectively). As depicted in the bar graph, the highest mean fluorescence peaked with AuNPs-d-PLL-PEG-FA.siRNA at 50 nM concentration compared to the low FL-1 levels of PSMA-siRNA lacking NPs. Interestingly, untreated PSMA-PC3 cells showed background levels similar to PSMA-siRNA at 25 nM. Moreover, AuNPs-d-PLL-PEG, at both 25 and 50 nM siRNA, showed no significant difference in fluorescence compared to control samples; the only difference was for cells incubated with AuNPs-d-PLL-PEG-FA.

### Uptake of fluorescein-siRNA conjugated NPs in PC-3 PSMA cells

**Figure 10:**
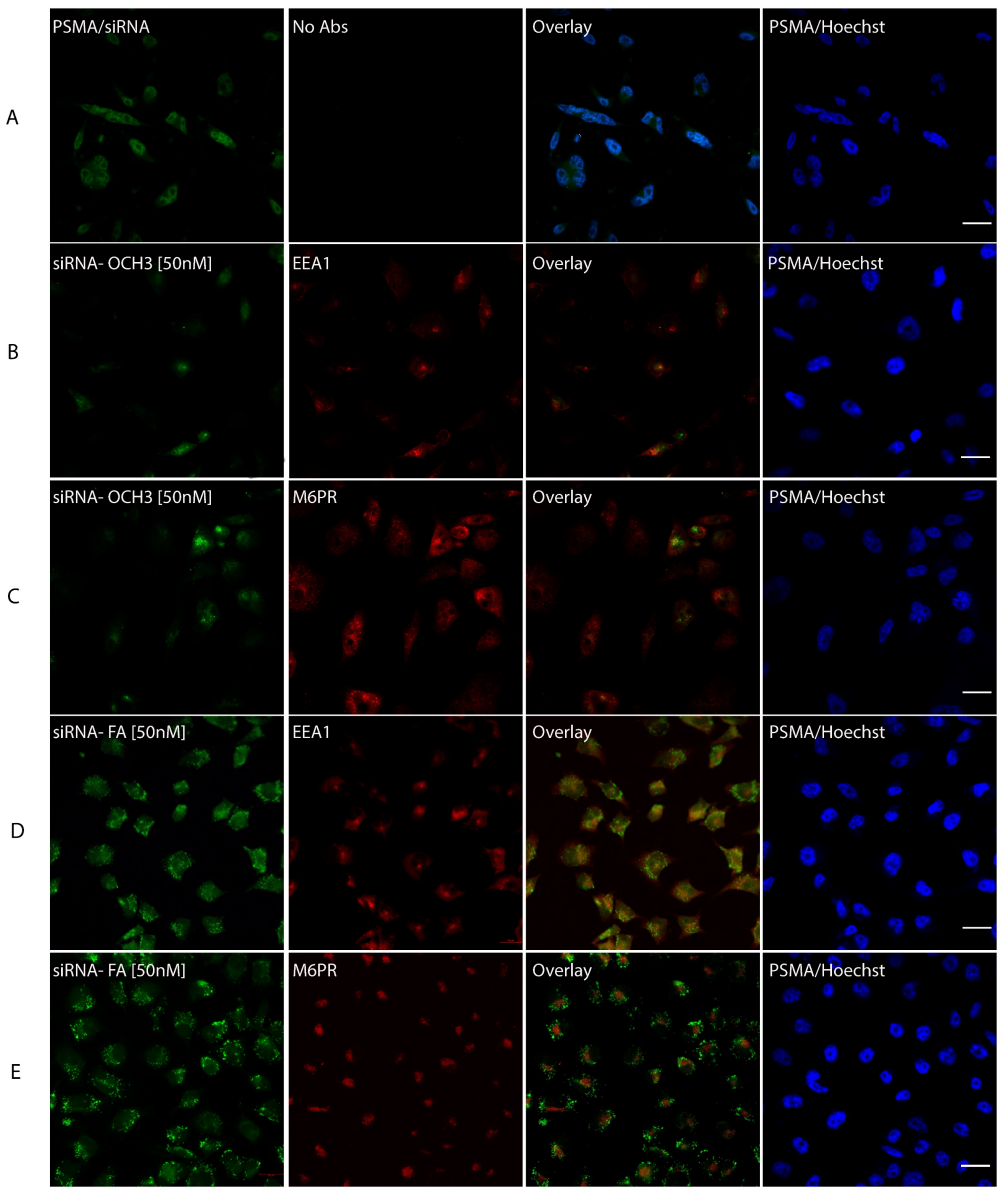
siRNA intracellular localization. PC-3 PSMA cells were incubated for 4 hours with siRNA.NP complexes, tagged with either -OCH3 (B, C) or -FA (D, E) at 37 °C. Panel A serves as a control, where PC-3 PSMA cells were incubated with 50 nM siRNA only. Cells were fixed in 100 % methanol at -20 °C for 5 mins and then stained with Hoechst and anti-early endosomal (EEA1 1/100) or lysosomal (M6PR 1/150) markers. The pattern of siRNA co-localization was visualized by Zeiss Confocal microscopy mainly from the overlay images. Only overlay from panel D showed dot-like structures with partial resemblance to early endosomes in almost 30% of the tested cells with siRNA-NP-FA-50 (D). Scale bar set at 20 µm (n = 5 /panel).

To decipher the exact subcellular localization of AuNPs-d-PLL-PEG-FA.siRNA 50 nM, cells were incubated with the NP complexes for 4 hours at 37 °C. Then they were fixed and stained with Hoechst, early endosomal marker (EEA1), or lysosomal marker (M6PR). AuNPs-d-PLL-PEG-FA.siRNA 50 nM was also visualized as dot-like structures dispersed in the cytoplasm, with remarkable perinuclear presence (Figure 10D and E, pseudo-green panels). Next, to determine the uptake pathway, we ought to merge the siRNA stain with EEA1 and M6PR stains. Interestingly, almost 30% of cells showed slight yellow zones indicating co-stains with the endosomal marker (Figure 10D and E, overlay). This overlap was only remarkable in cells treated with AuNPs-FA.siRNA and was absent in PSMA cells treated with AuNPs-d-PLL-PEG.siRNA 50 nM (Figure 10B and C, overlay).

**Figure 11:**
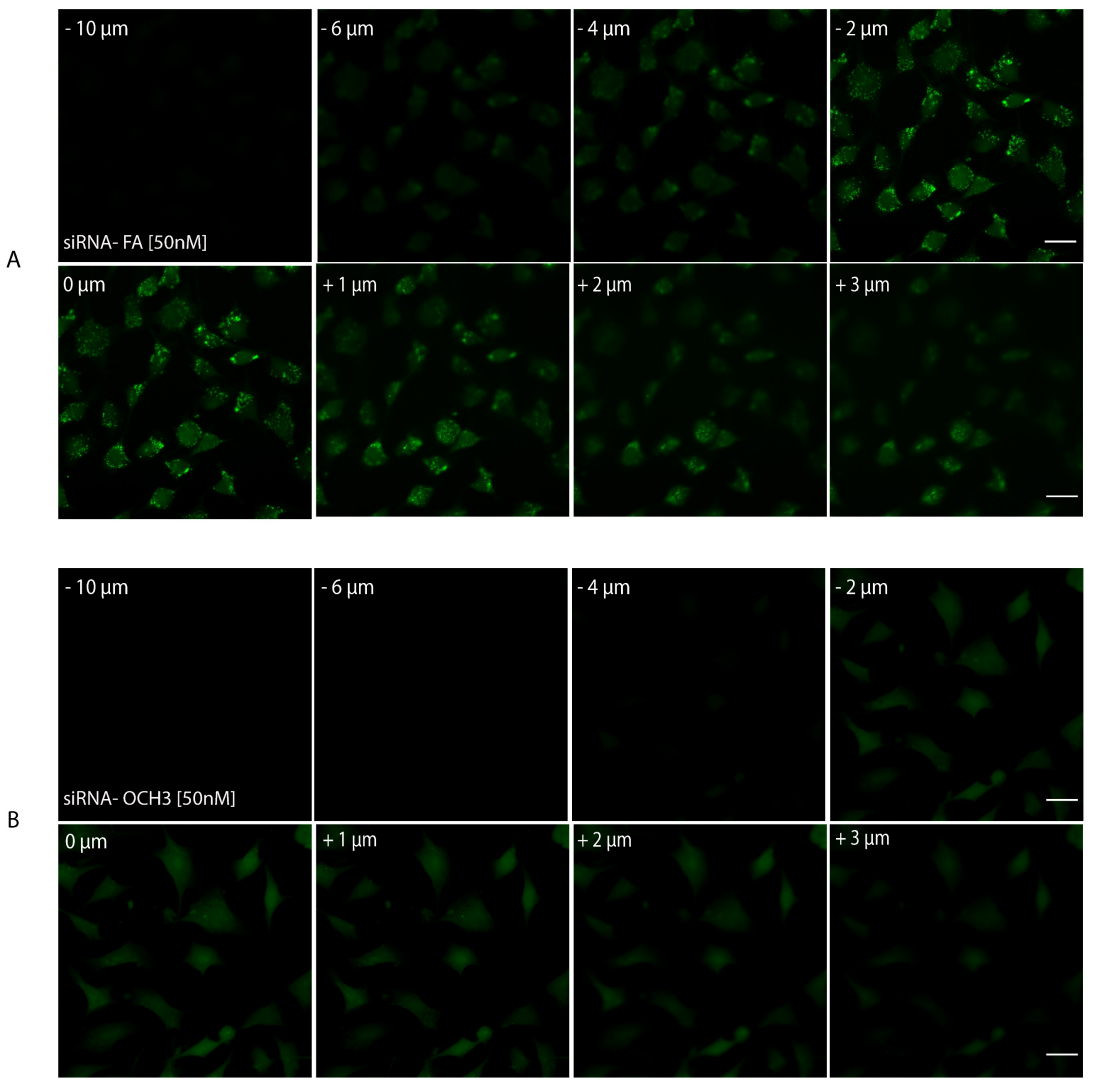
Z-stack imaging of PSMA cells treated with AuNPs-PEG.siRNA (50 nM) vs. AuNPs-FA.siRNA (50 nM). Eight out of 20 sections were chosen as representative sections at a set interval between -10 µm to +10 µm with positive and negative distances relative to the initial focal point of the image. All lasers had the same pinhole. The gain was set at 650 nm for FITC and 550 nm for Hoechst. There is a noticeable decrease in the FITC intensity of AuNPs-PEG.siRNA when compared to AuNPs-FA.siRNA. Furthermore, particles were only visible in sections ranging from -2 µm to + 1 µm in cells incubated with AuNPs-PEG-FA.siRNA (A); particles were absent in cells incubated with AuNPs-PEG.siRNA. The scale bar was set at 20 µm (n = 3).

Finally, as a last resort to confirm AuNPs internalization, Z-stack imaging was performed. Samples with AuNPs-d-PLL-PEG-FA.siRNA 50 nM and AuNPs-d-PLL-PEG.siRNA 50 nM were selected for a series of twenty consecutive images captured at focal distances ranging from - 10 µm to +10 µm. A set of eight images was chosen as a representative for both AuNPs-siRNA-FA 50 nM (Figure 11, panel A) and AuNPs-siRNA-OCH3 50 nM (Figure 11, panel B), respectively. A significant decrease in FITC intensity was noted for AuNPs-PEG.siRNA when compared to AuNPs-FA.siRNA. In addition, particles were only observed in sections ranging from - 2 µm to + 1µm in cells incubated with AuNPs-FA.siRNA, while they were completely absent in cells incubated with AuNPs-PEG.siRNA. Furthermore, a 2D render series of 1 frame/second was created to demonstrate this phenomenon (Video S1, data not shown). These findings indicate that our AuNPs are taken up by cells and reside in the cytoplasm.

## Discussion

Folate receptors are cellular membrane glycoproteins comprising three isoforms, namely FRα, FRβ, and FRγ 23. Folate receptors bind folic acid (FA) and structurally similar FA derivatives and mediate cellular delivery of these compounds. It has been established that folate receptors are overexpressed in many malignant tissues, including prostate cancer, while their expression is minimal in healthy tissues 21, 23. Targeted delivery via folate receptors can be utilized to increase efficacy and reduce the toxicity of medicinal agents for cancer therapy 22. In this study, FA was chosen as a targeting ligand and attached to thiolated poly (ethylene glycol) via an amide linkage to produce SH-PEG-FA 33. The latter was grafted onto positively charged AuNPs, synthesized via a chemical reduction in the presence of dendrigraft Poly-L-Lysine (d-PLL). The final cargo AuNPs-d-PLL-PEG-FA was found to successfully complex/deliver siRNA to prostate cancer cells. On the other hand, in a study published by Mbatha *et al.* FA modified poly(amidoamine) generation5 (PAMAM G5D) functionalized gold nanoparticles were used for siRNA luciferase gene slicing in folate receptor expressing HeLa-Tat-*Luc* cells 34. Their NPs were able to successfully complex and shield siRNA *in-vitro*. Additionally, the nano-formulations were non-cytotoxic and expressed formidable gene silencing capabilities 34. Furthermore, Chitosan and PEG_400_/PEG_2000_ functionalized AuNPs were also used to effectively deliver siRNA *in-vitro* for *c-MYC* silencing in an MCF-7 breast adenocarcinoma cell line 35. Such nanocomposites demonstrated a 90% decrease in MYC protein formation 35.

In our study, the strong positively charged nature of AuNPs facilitated the complexation with the negatively charged FAM siRNA via ionic charge interaction. In the case of AuNPs-d-PLL, the particles were shown to possess a high positive charge of 32 ± 1.3 mV. The latter allowed them to complex siRNA even at a low MR. As evident by gel electrophoresis, complexation is apparent at MR 10, but entirely achieved at MR 30. For AuNPs-d-PLL slightly neutralized with PEG-OCH3; the particles had the least positive zeta potential resulting in a fainter band at MR 10 when compared to AuNPs-PLL. However, as the MR of AuNPs-d-PLL-OCH3 to siRNA increased, complexation also increased until it was fully achieved at MR 30. Finally, concerning siRNA complexation with AuNPs-d-PLL-FA; the particles presented a zeta potential which is slightly less positive than AuNPs-d-PLL. The complexation pattern of these particles should be similar to AuNPs-d-PLL. Since FA is a larger molecule, it is believed to cause some steric interference, hindering the complexation with siRNA. The latter results in slightly more visible bands when comparing panels A and B at MR 10 14. Nonetheless, complete AuNPs-d-PLL-FA.siRNA complexation was achieved at MR 30. These results are aligned with J. Guo *et al*. previous findings, in which PEI-capped AuNPs behaved similarly, achieving complete siRNA complexation at minimal MR 20 for AuNPs-PEI-FA 14. Furthermore, results from another study by X. Luan *et al*. demonstrated full binding of siRNA with Au110-PEI-PEG_5000_ and Au110-PEI-PEG_5000_-AA at final MR40 36. These observations indicate that our NP complexes can easily and successfully complex siRNA for potential *in vitro* and* in vivo* cargo delivery. Moreover, flow cytometry analysis supplemented the electrophoresis readings to determine the optimal siRNA concentration for internalization. Results show that FA complexes, unlike OCH3 samples, bind to PC3-PSMA cells. There was no significant difference in the side scattering across the tested samples. This might be due to the negative surface charge of our NPs post siRNA complexation, causing slight repulsion with the negatively charged lipid bilayer of the cells 20. However, a clear increase in FL-1 intensity was present, especially for cells incubated with AuNPs-d-PLL-FA.siRNA at 50 nM. When incubated with AuNPs-d-PLL-PEG.siRNA at either 25 or 50 nM siRNA concentration, minimal fluorescence was detected (≤ 5%). This suggests that AuNPs-d-PLL-OCH3.siRNA do not bind to the FA receptors of the PC-3 PSMA cells. On the other hand, cells incubated with AuNPs-d-PLL-FA.siRNA (25 nM) presented a mean percent fluorescence of ~ 17%, indicating some interaction with PC-3 PSMA cells. Finally, cells incubated with AuNPs-d-PLL-FA.siRNA (50 nM) had the highest mean percent fluorescence, indicating that our AuNPs-FA complexes exhibit a specific binding capacity to FA receptors on the cell surface of PC-3 PSMA cells 37 and that a 50 nM concentration of siRNA is adequate for intracellular localization.

Laser scanning microscopy was challenging because it was difficult to pinpoint the exact method of AuNP uptake. PC-3 PSMA cells were incubated with AuNPs.FA.siRNA at 50 nM and AuNPs-OCH3.siRNA at 50 nM. Cells with AuNPs.FA showed vesicle-like structures while cells with AuNPs.OCH3 showed almost no siRNA signal. This is most likely an indication of particle internalization via the late secretory pathway. However, it was not enough to confirm that the particles reside in the cytoplasm. Therefore, cells were incubated again with AuNPs.FA.siRNA at 50 nM and AuNPs-OCH3.siRNA at 50 nM. Co-staining with EEA-1 1/100 and M6PR 1/150 was performed to identify whether the complexes are enclosed within early endosomes or lysosomes. After numerous attempts, we were unable to detect an overlapping siRNA signal for either EEA-1 or M6PR.

To undermine the exact location of our NPs, we supported the hypothesized observation that nanoparticles coated with positively charged and low pka amino groups, such as PEI or d-PLL, can undergo a process called the sponge effect and are most likely packaged in acidic vesicles 38. Through this process, cationic NPs bypass the cell surface lipid bilayer and enter acidic vesicles, where their amino groups sequester protons supplied by the proton pump, v-ATPase. Furthermore, the sequestered protons cause the pump to remain functioning, leading to the retention of chloride ions and water molecules. Finally, osmotic swelling ruptures the vesicles releasing the cationic NPs into the cytoplasm 38. This phenomenon has been repeatedly noted with NPs possessing a positively charged coating and is generally an acceptable hypothesis when dealing with positively charged polymers, like PEI and its derivatives 39.

## Conclusion and Future Perspectives

siRNA-based applications to treat cancer cells can only be successful if siRNA is properly shielded and specifically delivered to the target tumor site. Many nano-vehicles have been designed and reported in the literature. However, our proposed model of gold NPs features a new formulation. This includes not only a PEGylated shell, but also a dendrigraft of poly-L-lysine polymers to stabilize the NPs for better siRNA coating. To top it all off, the NPs were functionalized with folic acid solving the key problem of differentiating between healthy and malignant cells. The overexpression of folic acid receptors by proliferating tumor cells serves as natural ligands to signal site-specific binding of our nanocomposites. Although cellular uptake, intracellular localization, and siRNA mechanism of action dictate the functionality of NPs, we show here that the physicochemical properties of the nanocomposites are vital properties that govern the upstream biological behavior of all NPs. Thus, the reported nano-size, charge, and chemical coats in our NP formulations are sufficient to demonstrate minimal cytotoxicity, optimal siRNA-loading capacity, and specific binding to our tested prostate cancer cells.

Further progress in this direction will help monitor the efficiency of gene regulation in prostate or other types of cancer. Finally, translating the application of our tested NPs (AuNPs-d-PLL-FA.siRNA) from *in vitro* to a mouse model underpins the biocompatibility, biodistribution, and overall efficacy of using our nanocomposites as molecular cancer therapeutics.

## Supplementary Material

Supplementary Video 1 z stack.

## Figures and Tables

**Table 1:** Characterization of AuNPs-d-PLL, AuNPs-d-PLL-PEG, and AuNPs-d-PLL-PEG-FA via Dynamic Light Scattering and Zeta potential measurements. Readings were performed in triplicates in disposable folded capillary tubes (n = 3)

NPs	Z avg (d.nm)	PdI	Size I (d.nm)	Size N (d.nm)	Size V (d.nm)	Zeta Potential (mV)
AuNPs-d-PLL	118.8 ± 1	0.097 ±0.005	131 ± 1	89 ± 1	101 ± 30	32 ± 1.3
AuNPS-d-PLL-PEG	133.1 ± 0.9	0.083 ± 0.02	144.4 ± 0.3	114.13 ± 8	156.13 ± 0.3	21 ± 1
AuNPs-d-PLL-PEG-FA	125.5 ± 5	0.091± 0.033	143.3 ± 3	113.9 ± 10	155.06 ± 4.5	30 ± 0.9

**Table 2:** Characterization of AuNPs-d-PLL.siRNA 25 nM, AuNPs-d-PLL-PEG.siRNA 25nM vs. AuNPs-d-PLL-FA.siRNA 25 nM, via Dynamic Light Scattering and Zeta potential measurements. Readings were performed in triplicates in disposable folded capillary tubes. (All NPs at Mass:Ratio 25) (n = 3)

NPs	Z avg (d.nm)	PdI	Size I (d.nm)	Size N (d.nm)	Size V (d.nm)	Zeta Potential (mV)
AuNPs-d-PLL.siRNA 25 nM	123.5 ± 2.2	0.160 ± 0.043	132.43 ± 7	94.26 ± 2	137.26 ± 11	-21.13 ± 0.5
AuNPs-d-PLL-PEG.siRNA 25 nM	135 ± 4	0.094 ± 0.010	149 ± 3	111.6 ± 12.5	164.0 ± 8	-14.13 ± 4.87
AuNPs-d-PLL-PEG-FA.siRNA 25 nM	193.5 ± 1.9	0.113 ± 0.022	213.43 ± 7.53	187.33 ± 1.67	229.23 ± 35.3	-11.41 ± 3.78

**Table 3:** Characterization of AuNPs-d-PLL.siRNA 50 nM, AuNPs-d-PLL-PEG.siRNA 50 nM, AuNPs-d-PLL-FA.siRNA 50nM, via Dynamic Light Scattering (DLS) and Zeta potential measurements. Readings were performed in triplicates in disposable folded capillary tubes (All NPs at Mass: Ratio 25) (n = 3)

NPs	Z avg (d.nm)	PdI	Size I (d.nm)	Size N (d.nm)	Size V (d.nm)	Zeta Potential (mV)
AuNPs-d-PLL.siRNA 50 nM	139.6 ± 1.3	0.207 ± 0.023	168.7 ± 14.3	81.94 ± 7.94	78.97 ± 3.92	-23.6 ± 2
AuNPs-d-PLL-PEG.siRNA 50 nM	134 ± 1	0.146 ± 0.015	147.9 ± 6.1	94.47 ± 10	164.13 ± 20	-4.4 ± 1.4
AuNPs-d-PLL-FA.siRNA 50 nM	136 ± 1.5	0.207 ± 0.020	166.3 ± 14.3	78.6 ± 7.78	79.0 ± 2.69	-11.5 ± 1.5

## Abbreviations

AuNPs: gold nanoparticles
d-PLL: dendrigraft poly-l-lysine
DCC: *N, N'*-Dicyclohexylcarbidiimide
DMSO: Dimethyl sulfoxide
DLS: dynamic light scattering
EEA1: early endosomal marker
FA: folic acid
FTIR: Fourier-transform infrared spectroscopy
M6PR: lysosomal marker
MR: mass to ratio
λ_max_: maximum wavelength
NHS: N-hydroxy succinimide
PEG: poly (ethylene glycol)
siRNA: small interfering ribonucleic acid
SH- PEG_5000_-OCH3: thiol terminated poly (ethylene glycol) methyl ether
SH-PEG_5000_-FA: thiol terminated poly (ethylene glycol) folate
TEA: triethylamine
UV-Vis: ultra violet - visible
ξ: zeta potential
